# Case report: MELAS and T3271C mitochondrial mutation in an adult woman

**DOI:** 10.3389/fneur.2023.1179992

**Published:** 2023-07-27

**Authors:** Dong-hua Chen, Wei Li, Hai-shan Jiang, Chao Yuan

**Affiliations:** Department of Neurology, Nanfang Hospital, Southern Medical University, Guangzhou, Guangdong, China

**Keywords:** MELAS, T3271C mutation, peripheral nerve dysfunction, glucocorticoid therapy, case report

## Abstract

**Introduction:**

Patients with mitochondrial disorders always show neurological deficits. However, the diversity of clinical manifestations, genetic heterogeneity and threshold effect caused by maternal heredity make its diagnosis very challenging.

**Case presentation:**

A 30-year-old female presented to our neurology department with a recurrence of symmetrical weakness proximally in the lower extremities. Seven years ago, the patient had a sudden onset of persistent weakness in bilateral proximal lower extremities, along with elevated creatinine kinase (CK) and CK-MB. Given the diagnosis of Guillain-Barre syndrome, she was treated with high-dose glucocorticoid (GC) therapy at the local hospital and recovered. After admission to our hospital, laboratory analysis revealed elevated CK and alpha-hydroxybutyrate dehydrogenase in serum. Electrocardiography showed sinus tachycardia and left high ventricular voltage. Electromyography (EMG) and evoked potential (EP) suggested peripheral neurogenic damage of the upper and lower extremities with myogenic wear. Chronic inflammatory demyelinating polyneuropathy (CIDP) was initially considered, but neurological symptoms were not significantly improved with glucocorticoid shock therapy. An elevated level of lactate was found. The short-tau inversion recovery (STIR) axial magnetic resonance image (MRI) revealed mild hyperintensities, indicating muscle edema. Meanwhile, muscle biopsies suggested pathological changes in mitochondrial disorders (MIDs) and neuronal damage. Further mitochondrial genome analysis revealed a heteroplasmic m3271 T>C mutation in the mitochondrial tRNA-Leu gene (UUR). Collectively, the patient was finally diagnosed with mitochondrial disorder and apparently improved after the corresponding treatment to regulate energy metabolism.

**Conclusions:**

To our knowledge, it's the first report about MELAS with 3271 mutation that have only shown peripheral nerve motion impairment. Proximal weakness is also common in CIDP. In the context of this patient's experience, mitochondrial genome analysis provides an auxiliary criterion for differential diagnosis between MIDs and CIDP. In the meantime, we discussed the clinical effect of GCs on MIDs.

## Introduction

Mitochondrial diseases are caused by mutations in nuclear or mitochondrial genes, characterized by defects in oxidative phosphorylation (1). Mitochondrial diseases have diverse clinical manifestations, of which neurological deficits are the most common symptoms and probably make the greatest contribution to morbidity and mortality (2). However, the diversity of clinical manifestations, genetic heterogeneity and threshold effect caused by maternal heredity make its diagnosis very challenging. Mitochondrial encephalopathy with lactic acid and stroke-like episodes is one of the most common maternal mitochondrial diseases. Herein, we report the first patient of MELAS with T3271C mitochondrial mutation who presented only as impaired movement.

## Patient information

A 30-year-old female presented to our neurology department with **a** recurrence of symmetric**al** weakness proximally in the lower extremities. Seven years ago, the patient had a sudden onset of persistent weakness in the bilateral proximal lower extremities. At the time, she was able to stand with assistance and walk on her own. She then visited the local medical clinic for the first time at the age of 23. The only positive finding was the elevation of creatinine kinase (CK) (236 U/L) and CK-MB (29 U/L). She was then diagnosed with Guillain-Barre syndrome and largely recovered after high-dose glucocorticoid therapy.

## Clinical findings

Now the patient has been admitted to our hospital with a recurrence of neurological symptoms in the past 1 month. Neurological examination of the patient showed not only muscle weakness and hypotonia of extremities, but also muscle weakness of neck flexion [Medical Research Council (MRC) grade 4], elbow flexion (MRC grade 3) and knee extension (MRC grade 4). In addition, no tendon reflexes of the biceps brachii and triceps were found. No significant abnormalities were observed in cranial nerve examination, sensory system, autonomic nervous system, and meningeal irritation sign.

## Timeline

**Figure 1:**
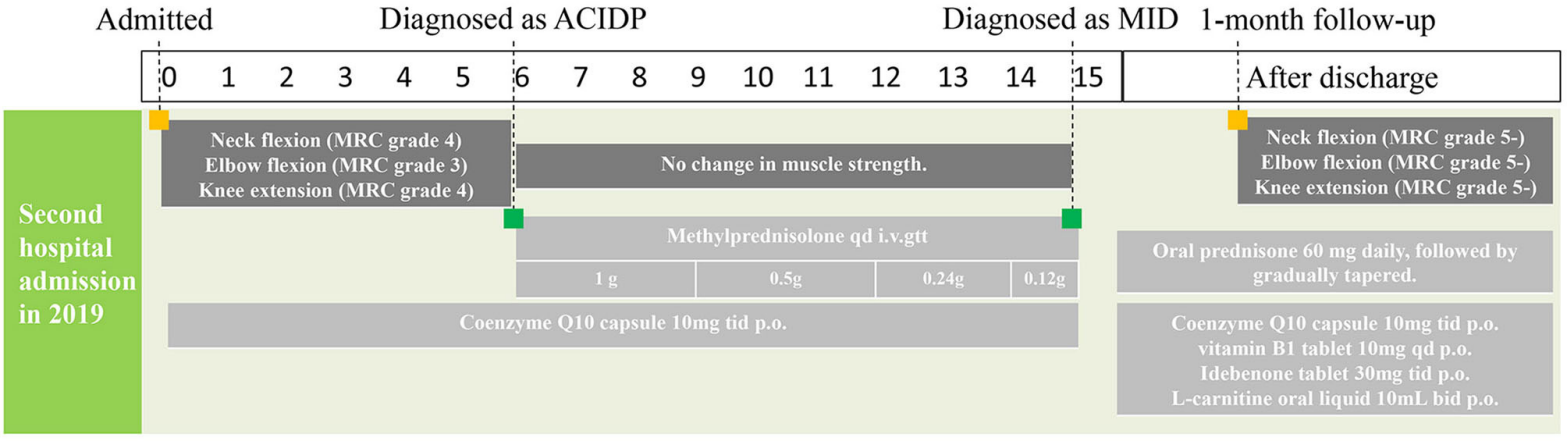
Timeline of this case report.

The timeline of this case report was shown below (Table 1; Figure 1).

**Table 1:** Timeline of this case report.

**Times**	**Events**
First hospital admission in 2012.	First appearance of muscle weakness in the lower extremities. Diagnosed with Guillain-Barre syndrome and treated with glucocorticoid therapy. Symptoms were largely recovered.
Second hospital admission in 2019 (Figure 1).	Recurrence of muscle weakness in the lower extremities. Misdiagnosed with Guillain-Barre syndrome and treated with glucocorticoid therapy. Diagnosed with mitochondrial disorder and treated with corresponding therapy to regulate energy metabolism. Symptoms were gradually recovered.

## Diagnostic assessment

**Figure 2:**
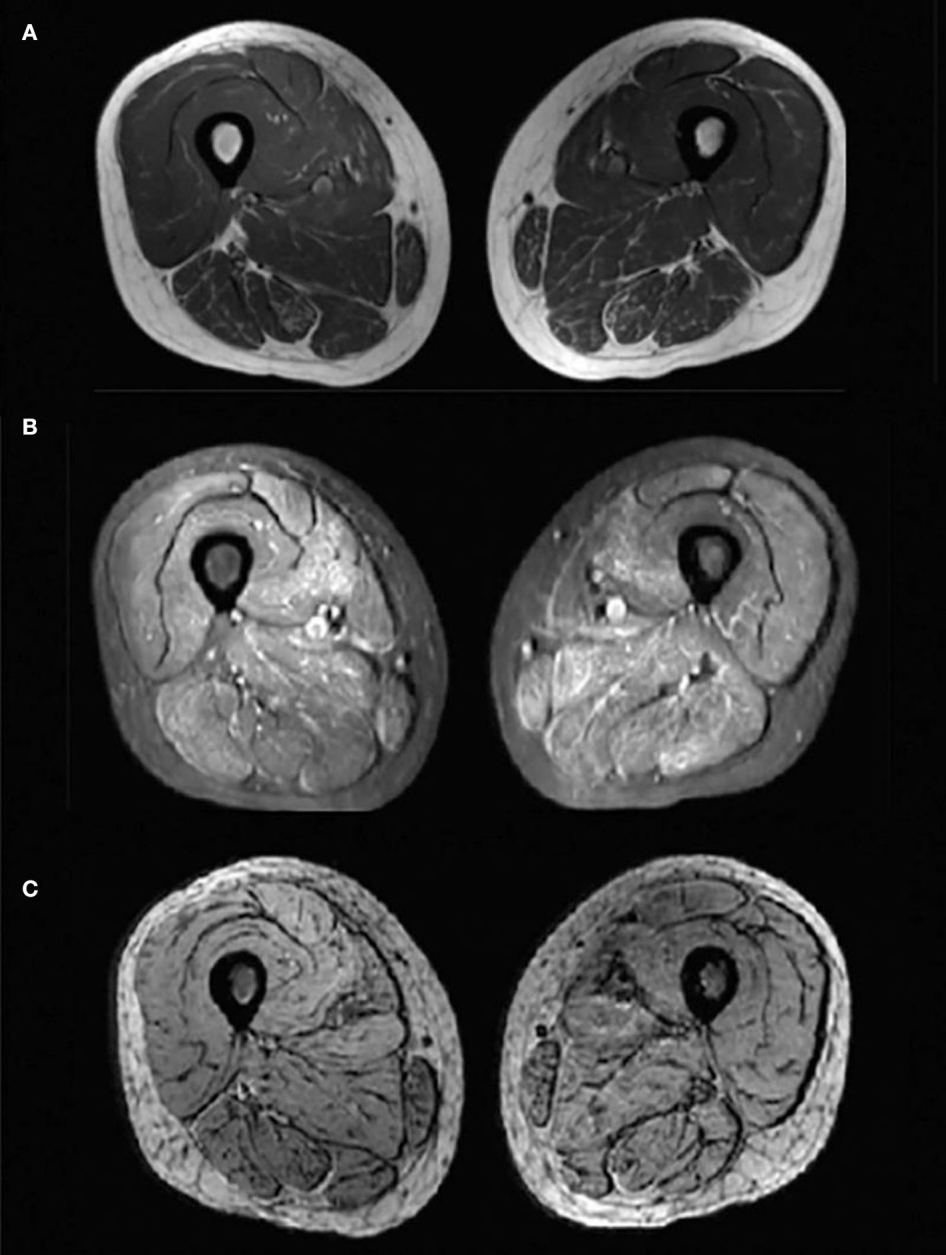
Magnetic resonance imaging (MRI) changes of bilateral thigh. Axial-T1-weighted MRI **(A)** showed hypointensity indicating no obvious fatty infiltration of the muscles and axial STIR MRI **(B)** showed slightly hyperintensities indicating mild muscle oedema. No hypointense structure was observed on axial SWI MRI **(C)**, indicating no angiogenesis.

**Figure 3:**
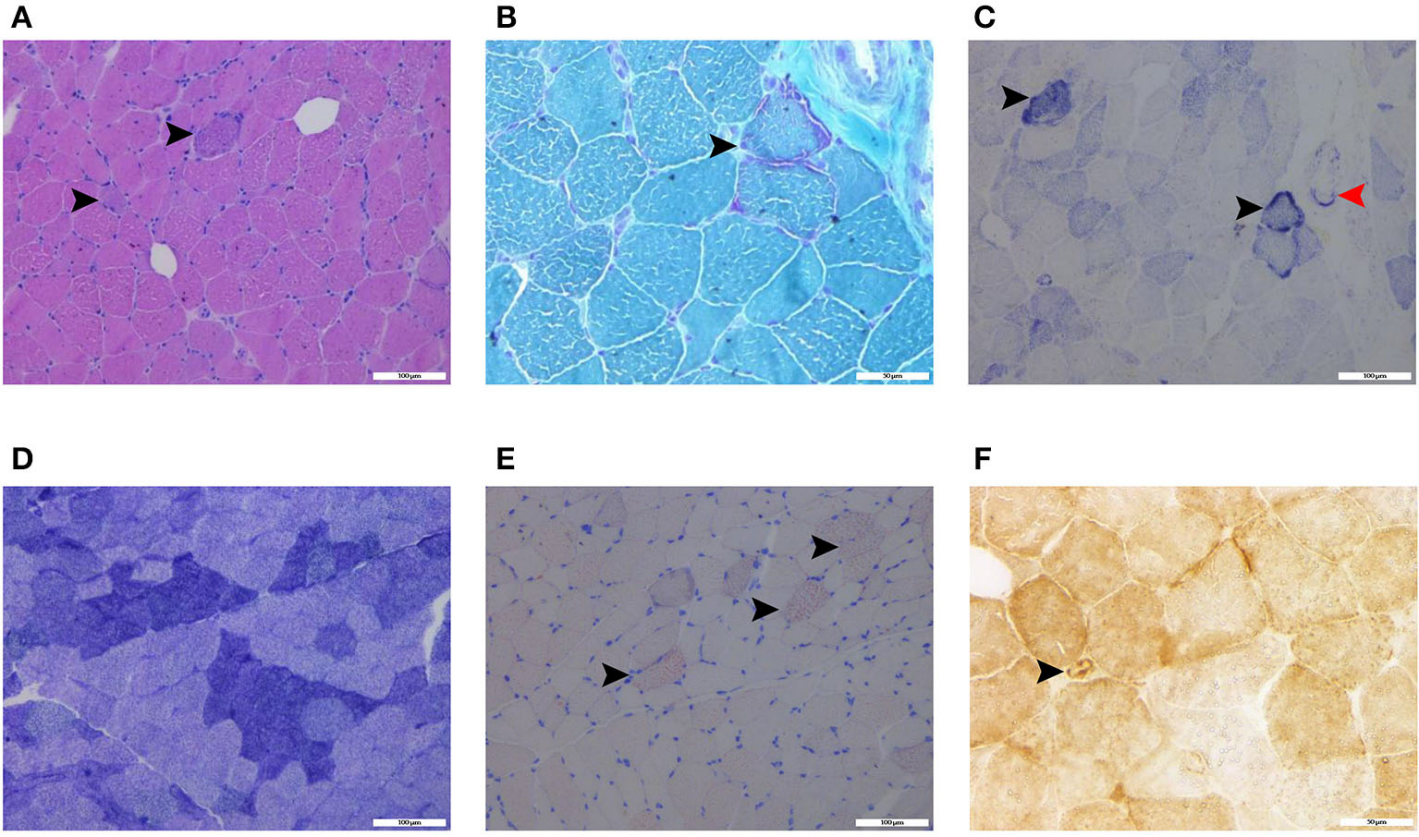
Histochemical stains of the thigh muscle biopsy specimens showed basophilic muscle fibers [**(A)**, haematoxylin and eosin stain, black arrows, scalebar: 100 μm], ragged-red fibers [**(B)**, modified Gomori trichrome stain, black arrow, scalebar: 50 μm], ragged-blue fibers [**(C)**, succinate dehydrogenase stain, black arrows, scalebar: 100 μm] and increased SDH activity in the vessels [**(C)**, succinate dehydrogenase stain, red arrow, scalebar: 100 μm], few grouping fibers [**(D)**, NADH-tetrazolium reductase, scalebar: 100 μm], increased fat droplets in some muscle fibers [**(E)**, Oil Red O stain, black arrows, scalebar: 100 μm], and COX-positive vessels [**(F)**, cytochrome c oxidase, black arrow, scalebar: 50 μm].

Laboratory analysis revealed elevated CK (246 U/L) and alpha-hydroxybutyrate dehydrogenase (alpha-HBDH) (232 U/L) levels in the patient. Results of other routine serologic investigations, cerebrospinal fluid (CSF) analysis, and autoimmune antibody titers, including peripheral neuropathy antibody spectrum in both serum and cerebrospinal fluid, were within normal limits. Electrocardiography revealed sinus tachycardia and left ventricular high voltage. Electromyography (EMG) and evoked potential (EP) suggested peripheral neurogenic damage of upper and lower extremities with myogenic damage. At the moment, the diagnosis of myopathy could not be ruled out, so we completed myositis autoantibody analysis and muscle magnetic resonance imaging (MRI). No myositis autoantibody was found. Under MRI scans (Figure 2), the characteristics of muscle lesions were not remarkable, and no obvious specific change in muscle group was found. However, there was edema in short-tau inversion recovery (STIR) sequences. Just then, a slight increase of blood lactic acid (8.7 mmol/L) was found in resting state, raising the suspicion of hereditary metabolic myopathy. Based on MRI finding, the tissue involved was then subjected to a muscle biopsy. The results (Figure 3) suggested pathological changes in MIDs and neuronal damage. So we thought MID might be the final diagnosis. In order to further clarify the diagnosis, mitochondrial DNA was carried out on the muscles involved and the results showed that there was a heteroplasmic m3271 T>C mutation in the mitochondrial tRNA-Leu gene (UUR), and the proportion of mutant mtDNA reached 87.4%.

## Therapeutic intervention

According to MID diagnostic criteria in 2002, the patient met two main diagnostic indexes and could be diagnosed as MID with MELAS (3). After the diagnosis was confirmed, coenzyme Q10, vitamin B1, idebenone, and L-carnitine oral liquid were given to improve the function of the oxidative respiratory chain and regulate energy metabolism (Figure 1).

## Follow-up and outcomes

After the treatment, the patient's symptoms had obviously improved and she could gradually stand and walk on her own. At 1-month follow-up, the muscle strength of neck flexion, elbow flexion and knee extension had recovered to MRC grade 5-/5.

## Discussion

MELAS is one of the most common maternal MIDs. Approximately 80% of MELAS patients harbor m3243A>G mutation, while ~7 to 15% of MELAS patients carry m3271T>C mutation (4–6). Peripheral nerve dysfunction alone was extremely rare for MELAS patients. To the best of our knowledge, this is the first report of patient with MELAS with T3271C mitochondrial mutation who presented only as motor impairment movement. We believe that this case is meaningful for further understanding of MELAs with m3271T>C mutation in clinical settings.

The treatment of MELAS aims to improve mitochondrial function and regulate energy metabolism. In addition, drugs that affect mitochondrial function should be used with caution. Meanwhile, the guidelines for the diagnosis and treatment of MIDs do not recommend the use of glucocorticoid (GC), but in fact, MELAS patients appear to benefit from the use of GCs (7). In line with them, the MELAS patient in our case showed an improvement in muscle strength after GC therapy in the early stages of the disease. One possible explanation for this is that GCs are involved in mitochondrial regulation by acting directly on mitochondrial DNA through the mitochondrially localized glucocorticoid receptor (GR) or indirectly through interaction with nuclear genes (8, 9). Worth mentioning, MELAS associated with pure motor CIDP, a type of atypical CIDP, couldn't be ruled out in our case (10), which may explain the effectiveness of GCs. If necessary, a nerve biopsy may be performed to confirm the diagnosis of CIDP. Close metabolic monitoring of the patient is crucial when the patient has worsening symptoms or new symptoms of other organs. Further, first-degree relatives of the patient were recommended for lactic acid detection and genetic testing.

In conclusion, MELAS is sometimes misdiagnosed as CIDP because of similar clinical manifestations, which may lead to the delayed treatment and missed diagnosis of the patient's family. If necessary, muscle biopsy and mitochondrial DNA analyses should be performed for differential diagnosis.

## Data availability statement

The original contributions presented in the study are included in the article/supplementary material, further inquiries can be directed to the corresponding authors.

## Ethics statement

Written informed consent was obtained from the individual(s) for the publication of any potentially identifiable images or data included in this article.

## Author contributions

D-hC, WL, H-sJ, and CY contributed to conception, design, acquisition of data, and analysis and interpretation of data. D-hC wrote the first draft of the manuscript. CY and H-sJ were responsible for revising manuscript. All authors contributed to read the manuscript and approved the submitted version.
